# The Processing of Lexical Ambiguity: Evidence from Child and Adult Greek

**DOI:** 10.1007/s10936-024-10063-y

**Published:** 2024-02-22

**Authors:** Maria Kaltsa, Despina Papadopoulou

**Affiliations:** 1 Department of Theoretical & Applied Linguistics, School of English, Faculty of Philosophy, Aristotle University of Thessaloniki, Thessaloniki, 54124 Greece; 2 Department of Linguistics, School of Philology, Faculty of Philosophy, Aristotle University of Thessaloniki, Thessaloniki, 54124 Greece

**Keywords:** Lexical ambiguity resolution, Homonyms, Sentential context, Greek, Child language

## Abstract

The aim of the study is to examine the effect of sentential context on lexical ambiguity resolution in Greek adults and typically developing children. Context and word frequency are factors that can affect lexical processing, however, the role of them has not been thoroughly examined in Greek. To this aim, we assessed sentence context effects in homonym meaning activation in monolingual speakers of Greek, children and adults, using a cross-modal priming paradigm. Additionally, all participants conducted a verbal working memory task and an inhibition task so as to examine whether the use of sentential context for lexical ambiguity resolution relates to age and/or cognitive processing capacity. The data analysis showed (a) major processing differences between adults and children due to ambiguity and sentential context, (b) children’s processing times affected by cognitive skills while adults’ processing unaffected, and (c) visual word recognition intact for all participants.

## Introduction

### The Linguistic cues in Lexical Ambiguity Resolution

The current study aims at examining the contribution of meaning frequency and contextual information in child and adult lexical ambiguity resolution via the exploitation of homonyms. Homonyms are lexical items with the same phonological and orthographic sequence but with two or more semantically unrelated meanings; for example, the word *bank* may refer either to land alongside a river or lake or a financial establishment (Lyons, 1977). Homonymy is a useful tool for the examination of meaning activation and selection and due to the fact that phonological and orthographic cues are constant and the frequency of each meaning measurable, it allows us to examine the true contribution of sentential context in ambiguity resolution. Previous work has indicated that word forms associated with multiple distinct meanings have been linked to increased processing load compared to unambiguous words on the basis of behavioral (Rodd et al., 2002), electrophysiological (Hagoort & Brown, 1994) and hemodynamic (Gennari et al., 2007) data. Ambiguity in written language in particular requires increased cognitive resources (Gadsby et al., 2008; Miyake, Just, & Carpenter, 1994). Event-related potential (ERP) studies show that noun/verb homographs in contexts providing syntactic, but not semantic, cues for ambiguity resolution show a sustained negativity over frontal–central scalp regions but when disambiguating semantic information is available, the frontal negativity is not evident (Federmeier et al., 2000; Lee & Federmeier, 2008, 2009)^1^. Earlier studies on lexical ambiguity resolution have identified sentential context, meaning frequency and word iconicity as some of the features that can affect lexical processing (Chen & Boland, 2008; Vu et al., 2000).

During language processing, the representation and maintenance of context information is critical for the successful comprehension of the stimuli, as the interpretation of words can vary significantly depending on their context of occurrence (Rodd et al., 2002, 2004). Data on lexical ambiguity resolution from adults show that during meaning competition for selection, sentential context influences the timing and/or degree to which the alternative meanings are activated. Andreou et al. (2009)^2^ tested 14 adult healthy native speakers of Greek to explore these parameters and found that both meanings of the ambiguous words were activated when the sentential context was not biased towards either meaning. Additionally, Andreou et al.’s findings revealed contextual effects, as an appropriately biasing sentential context facilitated the recognition of the meaning that was supported by it but not the non-favored meaning. Interestingly, the contextual effects were observed very early, when the inter-stimulus interval^3^ (ISI) was set at 0 ms and also later (ISI 750 ms).

It is important to note that children’s sensitivity to contextual cues during language processing can be traced even to children as young as 19 months old; Friedrich and Friederici (2005) identified a N400 effect for audio stimuli with a semantically anomalous word in an otherwise normal sentence. However, data on lexical ambiguity resolution from young school-aged children show that children do not exploit context similarly to adults (Booth et al., 2006). Booth et al. (2006) identified two patterns in their data: (a) sentential context effects increased from age 9 to 12, and (b) lexical-level effects decreased. In addition, earlier studies report facilitatory effects of congruent^4^, single-word context for children aged from 7 to 10 but inhibitory, incongruent single-word context effects for children aged 11 to 12 (Simpson & Foster, 1986; Simpson & Lorsbach, 1983). The difficulty to quickly and effectively integrate sentential context in lexical ambiguity resolution is also attested in monolingual children with developmental disorders (Henderson, Clarke & Snowling, 2011), even though there is no consensus in the literature regarding the locus of those difficulties (Brock et al., 2008; Hahn et al., 2015; Norbury, 2005).

A key factor in lexical ambiguity appears to be the relative frequency of the word’s alternative meanings. There are balanced ambiguous words, in that the two meanings of these words have approximately equal association strengths to the ambiguous word form. An example of a balanced ambiguous word in English would be the word *mold*; referring either to a container used to give shape to a liquid material or fungus with similar frequency for both meanings (example from Klepousniotou et al., 2012). Some ambiguous words, on the other hand, are biased, since one meaning is much more strongly associated with the word form and is referred to as dominant meaning, while the alternative meaning is the subordinate one. For example, the word *ball* in English has a dominant meaning referring to a spherical object that is kicked or thrown in a game and a subordinate meaning referring to dancing (example from Klepousniotou et al., 2012). Several studies have indicated that the more frequent, dominant meaning of an ambiguous word is activated more quickly than less frequent, subordinate meanings in neutral sentence contexts (Dopkins et al., 1992; Lucas, 1999; Sereno, Brewer, & O’Donnell, 2003; Simpson & Krueger, 1991). In the case of the *ball* paradigm, a neutral sentential context would not be related to either meaning as in *the doctor held the ball* which does not bias to either direction as *the football player kicked the ball* would. Duffy, Morris and Rayner (1988) report significant processing cost in reading times (RTs) on a biased ambiguous word when the sentential context biases towards the word’s subordinate meaning, but no such effects for balanced ambiguous word (see also the discussion in Vuong & Martin, 2011).

The subordinate bias effect has been demonstrated in a number of studies, which showed that RTs for ambiguous words are longer compared to those for unambiguous control words matched in frequency (Binder, 2003; Kambe et al., 2001; Rayner et al., 2006; Sereno, O’Donnell, & Rayner 2006). For the subordinate bias effect, however, to be evident two conditions need to be met: (a) the homonym must be strongly polarized, meaning that there is a more pronounced bias ratio with subordinate meanings retrieved only about 10% of the time in word association tasks, and (b) the frequency of the control unambiguous word needs to be matched to the homograph’s form frequency, that is the sum of all meaning frequencies (Binder, 2003; Binder & Rayner, 1998; Kambe et al., 2001; Pacht & Rayner, 1993; Rayner et al., 2006; Sereno, O’Donnell, & Rayner, 2006; Sereno et al., 1992). Meanwhile, research in younger speakers suggests that children are not sensitive to the relative frequency of each meaning of ambiguous lexical items (Booth et al., 2006; Simpson & Foster, 1986).

### Lexical Access Models for Ambiguity Resolution

To accommodate the variation in findings with regard to child and adult monolingual data a number of lexical access models have been put forward. According to the reordered access model both meanings of ambiguous words are activated and remain in competition till ambiguity is resolved (Duffy et al., 2001). In light of this model, initial access of word meaning is not immune to contextual effects; for the biased ambiguous words even in the presence of disambiguating context that biases towards the subordinate meaning, the dominant meaning will be activated. Meanwhile the context-sensitive/selective access account proposes that the dominant meaning is not activated if the context is sufficiently constraining towards a subordinate meaning; possible constraints for this processing model can be frequency, type of context and strength of context (Kellas et al., 1995; Vu et al., 2000, 2003).

Another lexical access model for ambiguity resolution is the dual mechanism account by Gernsbacher and St. John (2001), according to which there is a first mechanism described as the bottom-up, frequency-weighted activation of all homonym meanings and a second mechanism as the top-down^5^ suppression of contextually irrelevant meanings. Gernsbacher’s and St. John’s (2001) proposal also makes a reference to variability due to reading skills; with less skilled readers having less efficient mechanisms for suppression of the unintended meaning. Yet children’s data from linguistic ambiguity resolution show some disparity in the use of bottom-up and top-down cues depending on their age and it remains an open question which type of lexical access account could have higher explanatory value for their course of processing (see Rabagliati et al., 2013; Snedeker, 2013; Snedeker & Trueswell, 2004; Snedeker & Yuan, 2008; Trueswell & Gleitman, 2007).

### The role of Cognitive Capacity

As already presented above, the adult lexical ambiguity literature emphasizes the relative frequency of alternative meanings as well as the interaction between contextual bias and frequency (Binder & Rayner, 1998; Chen & Boland, 2008; Vu et al., 1998); however, even among healthy adults there is some variability in their performance that appear to relate to working memory (WM) and reading skills. Miyake, Just and Carpenter (1994) point that an individual’s working memory capacity relates to the attention and activation a person can assign to maintain lexical information activated during processing; specifically, their data suggest that, while individuals with low WM skills activate the most frequently accessed dominant meaning, individuals with high WM skills could maintain both meanings activated even after an eight-word delay. Gunter et al. (2003) also found that high WM capacity leads to higher efficiency in inhibiting alternative meanings of ambiguous words. Moreover, Gadsby et al. (2008) used a priming task with stimulus words arranged into word triplets with each triplet including two prime words followed by a target to examine congruent relationships (both the context and the target related to the same meaning of the homograph) and incongruent relationships (context and target related to different meanings of the homograph). They found evidence that individuals with high working memory spans exhibited a pattern of priming for congruent conditions and a lack of positive priming for incongruent conditions when processing homographs. Apart from cognitive resources, literacy skills also appear to affect performance. Gernsbacher and colleagues (Gernsbacher, 1993; Gernsbacher et al., 1990) showed that skilled readers can quickly suppress the contextually inappropriate meaning of an ambiguous word but less skilled readers are less effective.

Given that WM and reading skills appear to affect steady state grammars, when looking into developing grammars, it is essential to weigh the contribution of the cognitive processing capacity since it is itself under development. Literature points to the bidirectional relationship of language comprehension and working memory (see Payne, Kalibatseva, & Jugers, 2009; Seigneuric et al., 2000; Oakhill et al., 2003). Diamond (2006) cites the years from 5 to 11 as marking substantial improvements in cognitive flexibility, working memory, and processing speed. The failure to engage in top-down processes, which is often reported in child data, may be related to the current level of cognitive resources that need to be allocated to process language. Khanna and Boland (2010) Examined lexical ambiguity resolution in children aged 7 to 10 years and adults testing the impact of sentential and single-word contexts in cross-modal naming paradigms. Their data showed that when a sentential context was provided only older children and adults showed priming for context-appropriate targets, whereas when a single-word context was provided all groups showed contextual sensitivity. Additionally, their analysis highlighted that mature executive function abilities as measured via working memory span and inhibition skills were found to be associated with greater contextual sensitivity. Similarly, Coch and Holcomb (2003) advocate that younger and less skilled readers exhaust their cognitive resources while reading, leaving inadequate resources for the context integration process; consequently, it is necessary to factor in the role of cognitive skills such as working memory and inhibition which are necessary for top-down processing (Bunge et al., 2002; Casey et al., 2005; Protopapas et al., 2007).

### Research Questions

The present study employs homonyms to explore the factors that affect lexical ambiguity resolution in Greek, an area largely understudied for typically developing, but also developed Greek speakers (for English see Khanna & Boland, 2010). More specifically, we address the following research questions:

- whether meaning frequency and context affect Greek adult and child lexical ambiguity resolution,
- whether language external factors such as age and cognitive skills (inhibition and verbal working memory) play a role in Greek adult and child lexical ambiguity resolution.

To do so, we assessed sentence context effects in homonym meaning activation in monolingual speakers of Greek, children and adults, using a cross-modal priming paradigm, similar to the one employed by Andreou et al. (2009); that is with a sentential context provided in audio and a visual target that related to either dominant or subordinate meaning presented in detail in the following section. Note that factoring in the different results attested by the Khanna and Boland (2010) study on sentence and single-word contexts, we opted for sentential contexts. To tease apart maturational differences in sentential context integration from cognitive load differences related to reading skills, we have selected an experimental paradigm that includes little reading; namely a cross-modal priming task with the prime being an audio stimulus of a sentence and the visual target a single short word.

## Method

### Pre-tasks

The main experiment of the current study is a cross-modal priming task that examines lexical ambiguity resolution using homonyms in neutral and biasing contexts. Homonyms and control items that were included in the experimental design were selected with the use of three pre-tasks. The participant recruitment process across those pre-tasks is stratified random sampling; the stratified variables in the population were age, sex and educational level. First pre-task A was administered; on the basis of the results of pre-task A, the second pre-task was developed and then pre-task C followed. Specifically, to select the appropriate homonym items, we employed the data of pre-task A, to select the target items for the visual word recognition we employed pre-task B and to select the appropriate control items we employed the data of pre-task C. These pre-tasks were necessary, as there are no standardized lists of ambiguous words in Greek, as there are in English. The minimum requirement in terms of sample size for the pre-tasks was set to 50 participants and was satisfied in all cases.

Pre-task A tested the competing meaning frequencies of ambiguous items. Participants were asked to provide all possible meanings for 142 ambiguous noun words in Greek. 50 adult native speakers participated in the task (F: 27; M: 23 | Age M: 30; Age Range: 21–62). On the basis of the analysis 30 homonyms were selected for the main experiment with a high frequency dominant meaning (M: 74.8%; Range: 56–86%) and a low frequency subordinate meaning (M: 38.4%; Range: 32–46%); the exclusion criteria for the ambiguous words were (a) both meanings with the same frequency, and (b) frequency above 86% or below 32% so as to avoid highly polarized items.

Pre-task B aimed at identifying the target items for the visual word recognition that are strongly related to the homonyms for each of the two meanings. 84^6^ adult native speakers of Greek participated in the task (F: 48; M: 36 | Age M: 24; Age Range: 18–39). On the basis of the analysis, the most frequently first associated target words to each meaning were selected. All visual targets have the same word length and frequency across conditions; N letters M: 6.5 (SD: 1.7); N syllables M: 2.9 (SD: 0.9) and similar frequency according to Hellenic National Corpus (HNC: http://corpus.ilsp.gr).

Pre-task C is a word familiarity task that was used to verify that homonyms and control words were of similar frequency. Participants were asked to rate items on a 5-point scale with 1 allocated to least frequent items and 5 to most frequent items. 52 adult native speakers of Greek participated in the task (F: 28; M: 24 | Age M: 26; Age Range: 19–45). The analysis confirmed that homonyms (M: 3.8, SD: 0.4) and control items (M: 3.9, SD: 0.6) selected are of the same frequency.

### Experimental Design of Cross Modal Priming Task

For the main component of the study a cross-modal priming task was developed in order to examine the access of multiple meanings of ambiguous lexical items in neutral and biasing contexts. We opted for relatively equibiased homonyms for ambiguity resolution so as to minimize frequency-driven meaning selection that is found in highly polarized ambiguous words and thus avoid any subordinate bias effect in our data. The types of sentential context that we examined are dominant meaning biased, subordinate meaning biased and not related to either meaning. The cross-modal priming experiment was a speeded lexical decision task with the prime being an audio stimulus (homonym or control word) that appeared at the end of sentences biasing the dominant, the subordinate or neither meaning and the visual target words related to either the dominant or the subordinate meaning of the homonym examined. Participants were instructed that they would listen to a sentence and subsequently a visual target would appear on the screen; their task was to indicate whether the visual target was a word or not in Greek by pressing one of the two pre-specified buttons on the keyboard. The visual targets that appeared in the experimental conditions were selected on the basis of the outcomes of pre-task B. Table 1 exemplifies the experimental paradigm:

**Table 1:** Experimental Paradigm

Audio Prime Homonym: αγγείο [aˈgio] (pot, vessel) Control: χαρτί [xarˈti] (paper)	Visual Target	
	dominant meaning	subordinate meaning
*dominant meaning-biased context* ˈEvale ˈliɣo neˈro sto aˈgio/xarˈti. Έβαλε λίγο νερό στο αγγείο/χαρτί. ‘He poured some water into the pot/paper.’		
*subordinate meaning-biased context* O ɣiaˈtros ˈtripise to aˈgio/xarˈti. Ο γιατρός τρύπησε το αγγείο/χαρτί. ‘The doctor cut through the blood vessel/paper.’	ˈvazo βάζο ‘vase’	ˈema αίµα ‘blood’
*Unrelated context* ˈIθele na ði to aˈgio/xarˈti. Ήθελε να δει το αγγείο/χαρτί. ‘She/he wanted to see the pot/vessel / paper.’		

In the paradigm, the homonym *a*ˈ*gio* bears the dominant meaning ‘pot’ and the subordinate meaning ‘vessel’ and appeared in three different contexts; one that biases towards the ‘pot’ meaning (ˈ*Evale* ˈ*liɣo ne*ˈ*ro sto a*ˈ*gio.* He poured some water into the pot.); one that biases towards the ‘vessel’ meaning (*O ɣia*ˈ*tros* ˈ*tripise to a*ˈ*gio.* The doctor cut through the blood vessel.); and an unrelated, neutral context (ˈ*Iθele na ði to a*ˈ*gio*. She/he wanted to see the pot/vessel.). The audio was followed by the visual targets *ˈvazo* ‘vase’ and *ˈema* ‘blood’. The same set of sentential contexts and visual targets were included in the control conditions with the homonym word being replaced by the control word *xarˈti* ‘paper’.

In this cross-modal priming task 30 homonyms were examined along with the same number of control items for all sentential contexts. Each homonym and control item appeared in 6 conditions that led to the development of 360 experimental items and 360 fillers. The length of all sentences that included the primes was 5–7 words (M: 5.6; SD: 0.6) and it did not statistically differ among conditions tested. For the filler items, visual targets were either illegal non-words with a phonotactic violation in the consonantal cluster of the first syllable (e.g. *ˈzlota*) or pseudo-words that do not violate Greek phonotactic rules (e.g. *ˈkreza*) so as to support the lexical decision component of the task. Given Andreou et al. (2009) findings on the absence of ISI effects, ISI was set to 0ms in the task. Stimuli were presented using E-Prime 2.0 software (Psychology Software Tools, 2012) and items were equally divided and randomized in six experimental versions, so that each version contained all types of primes and visual targets, while the same visual target and the same audio prime were never encountered more than once in each version. We administered the six experimental sessions to every participant in the study. The task was administered in a quiet room; 5 practice items were also included at the beginning of each session so that participants familiarize themselves with the experimental procedure. The duration of each session was approximately 15 min.

### Research Hypotheses

Based on previous research on adult lexical ambiguity resolution (see Binder, 2003; Rayner et al., 2006; Rodd et al., 2004; Sereno, O’Donnell, & Rayner, 2006; Vu et al., 2000; Vuong & Martin, 2011), we expect the meaning frequency of the ambiguous words and the sentential context to affect word recognition. Lexical processing in children, on the other hand, is expected to be slowed down by contextual integration, since previous studies indicated difficulties with context integration in word recognition and limited sensitivity to the frequency of competing meanings (see Booth et al., 2006).

Regarding the relation between lexical processing and cognitive skills and considering that adults are skilled readers, we expect correlations between working memory capacity and lexical decision times and also between inhibition and effective integration of the sentential context cues in word recognition to be more pronounced in the child dataset (for skilled reading effects see Gernsbacher, 1993; Gernsbacher et al., 1990; for children see Bunge et al., 2002; Casey et al., 2005; Gunter et al., 2003; Khanna & Boland, 2010; Protopapas et al., 2007).

### Participants

20 children (F:10, M:10) aged 10;4 to 11;3 years old (M:10;8, SD:0;3) participated in the main study^7^. Their cognitive screening included (a) the Raven’s Progressive Matrices Test (Raven, 2003) as a well-validated measure of basic cognitive functioning for non-verbal intelligence (M: 30.65; SD: 2.27); no child participant was eliminated on the basis of their Raven’s Matrices performance, (b) the Digit Span Backwards Recall of the Wechsler Adult Intelligence Scale (WAIS-IV, 2008) as a measure of verbal working memory (M: 14.85; SD: 4.61), and (c) the Nonverbal Stroop Card Sorting Test (Roid & Koch, 2017) as an inhibitory control measure (M: 47.85; SD: 2.11). The adult participants’ age (N: 20; F:11, M:9) ranged from 23 to 38 years old (M:29, SD:5), all of them were university graduates (Μ: 19.1 years of education) and their cognitive screening included the Digit Span Backwards Recall of the Wechsler Adult Intelligence Scale (WAIS-IV, 2008) as a measure of verbal working memory (M: 23.6; SD: 4.55) and the Nonverbal Stroop Card Sorting Test (Roid & Koch, 2017) as an inhibitory control measure (M: 39.05; SD: 14.78). None of the participants had a hearing or visual impairment issue or any other language related difficulty; relevant information was obtained when consent form was signed in a questionnaire format and school records were also advised during the recruiting process of child participants.

### Data Analysis

Our design involved two dependent variables, accuracy and Lexical Decision Times (LDTs). To analyze the data^8^, we performed two separate analyses for each dependent variable. More specifically, we ran repeated measures analyses of variance (ANOVA), with Bonferroni correction in the Post Hoc analysis, with Ambiguity (homonyms vs. controls), Frequency (dominant meaning high frequency vs. subordinate meaning low frequency) and Context (dominant meaning bias vs. subordinate meaning bias vs. unrelated) as the within subjects variables and Age (children vs. adults) as the between subjects variable. When the interaction among all factors was significant, we performed follow-up *t*-tests in each group in order to further explore the locus of the interaction. In the follow-up *t*-tests we firstly explored in which conditions we get a facilitation (positive priming, faster LDTs for visual targets following homonyms than control words) or an inhibition (negative priming, faster LDTs for visual targets following control words over homonyms) effect. We further explored (a) the effect of meaning frequency by comparing the priming effect for dominant with that for subordinate meaning visual targets in each context condition and (b) the effect of context by comparing the priming effect for the unrelated with the one for the dominant/subordinate context in each frequency meaning.

## Results

### Lexical Decision Times (LDTs)

Firstly, we present the online data, lexical decision times (LDTs), on the visual targets. LDTs were screened for extreme values and outliers. Outliers were defined as LDTs above or below 2 standard deviations from the mean LDT in each condition separately per subject and item. Outliers were replaced with the mean LDT for each condition per subject and item once this value was removed. This procedure affected 2.75% of the data. To analyze the processing data, we excluded incorrect responses and we performed a repeated measures analysis of variance (ANOVA) with Bonferroni correction in the Post Hoc analysis, with Ambiguity (homonyms vs. controls), Frequency (dominant meaning high frequency vs. subordinate meaning low frequency) and Context (dominant meaning bias vs. subordinate meaning bias vs. unrelated) as the within subjects variables and Age (children vs. adults) as the between subjects variable; follow-up *t*-test comparisons were conducted where necessary. Table 2 shows the LDTs in visual word recognition per condition for both groups.

**Table 2:** Processing Data [LDTs in msec, SDs in parentheses]

Audio Prime	Visual Target	Experimental Item	Children	Adults
unrelated context	dominant meaning	Homonym	1017 (335)	572 (119)
		Control	948 (270)	594 (154)
	subordinate meaning	Homonym	1227 (380)	574 (101)
		Control	953 (323)	573 (109)
dominant meaning context bias	dominant meaning	Homonym	1346 (225)	610 (158)
		Control	1082 (324)	578 (121)
	subordinate meaning	Homonym	875 (275)	571 (150)
		Control	1105 (344)	582 (104)
subordinate meaning context bias	dominant meaning	Homonym	1296 (360)	586 (105)
		Control	964 (300)	571 (110)
	subordinate meaning	Homonym	1320 (293)	616 (150)
		Control	1073 (310)	577 (105)

The analysis showed a main effect of Age [*F*_*1*(1, 38)_ = 64.590, *p* < .001, *η*^2^_*p*_ = 0.630; *F*_*2*(1, 54)_ = 2.456, *p* < .001, *η*^2^_*p*_ = 0.978] with adults processing times being considerably faster than those of children (adults: 584 < children: 1103), a main effect of Ambiguity [*F*_*1* (1, 38)_ = 15.349, *p* < .001, *η*^2^_*p*_ = 0.288; *F*_*2*(1, 54)_ = 193.649, *p* < .001, *η*^2^_*p*_ = 0.782] with homonyms processing being highly costly (homonyms: 889 > controls: 797), a main effect of Context [*F*_*1* (2, 76)_ = 23.054, *p* = < 0.001, *η*^2^_*p*_ = 0.378; *F*_*2*(2, 108)_ = 115.492, *p* < .001, *η*^2^_*p*_ = 0.681] with faster LDTs for unrelated context and slower LDTs for subordinate context bias (unrelated: 806 < dominant context bias: 842 < subordinate context bias: 882; all pair comparisons: *p* < .001) and an interaction among all factors [*F*_*1* (2, 76)_ = 11.514, *p* < .001, *η*^2^_*p*_ = 0.233; *F*_*2*(2, 108)_ = 69.031, *p* < .001, *η*^2^_*p*_ = 0.561].

The analysis of the adult data revealed only a few significant comparisons. Specifically, with regard to ambiguity, a facilitation effect with control words being slower than homonyms was attested only for dominant meaning visual targets in unrelated contexts [*t*_*2*_ (27) = -3.207, *p* = .003]. An inhibition effect with control words processed faster than homonyms was found for dominant meaning visual targets in the dominant meaning bias contexts [*t*_*2*_ (27) = 4.919, *p* < .001] and for subordinate meaning visual targets in subordinate contexts [*t*_*2*_ (27) = 7.480, *p* < .001]. With reference to frequency effects, the analysis shows that the dominant meaning is facilitated over the subordinate meaning in unrelated [*t*_*2*_ (27) = -2.473, *p* = .020] and in subordinate meaning bias contexts [*t*_*2*_ (27) = -2.822, *p* = .009]. Interestingly, the opposite effect is obtained in dominant meaning bias contexts, as facilitation was found for the subordinate over the dominant meaning [*t*_*2*_ (27) = 4.717, *p* < .001]. Turning to the effects of context, we found that the dominant or the subordinate bias context did not facilitate the recognition of the homonym in either meaning bias. On the contrary, the unrelated context enhanced the facilitation (a) of the dominant meaning in dominant and subordinate bias contexts [dominant bias context: *t*_*2*_ (27) = -5.848, *p* < .001; subordinate bias context: *t*_*1*_ (19) = -2.048, *p* = .055; *t*_*2*_ (27) = -3.474, *p* = .002] and (b) of the subordinate meaning in the subordinate bias context [*t*_*2*_ (27) = -5.017, *p* < .001].

Further analysis of the child dataset revealed that ambiguity adds processing cost across conditions [dominant meaning visual target – dominant context bias: *t*_*1*_ (19) 3.452, *p* = .003; *t*_*2*_ (27) = 7.073, *p* < .001; dominant meaning visual target – subordinate context bias: *t*_*1*_ (19) = 4.365, *p* < .001, *t*_*2*_ (27) = 14.625, *p* < .001; dominant meaning visual target – unrelated context: *t*_*2*_ (27) = 2.320, *p* = .028; subordinate meaning visual target – subordinate context bias context: *t*_*1*_ (19) = 3.599, *p* = .002, *t*_*2*_ (27) = 8.689, *p* < .001; subordinate meaning visual target – unrelated context: *t*_*1*_ (19) = 4.005, *p* = .001, *t*_*2*_ (27) = 12.312, *p* < .001] with one exception; a facilitation effect for homonyms over control words was obtained only with subordinate meaning visual targets in dominant bias contexts [*t*_*1*_ (19) = -3.564, *p* = .002, *t*_*2*_ (27) = -9.172, *p* < .001]. Regarding frequency, the dominant meaning visual targets were significantly more primed than the subordinate meaning ones in unrelated contexts [*t*_*1*_ (19) = -2.994, *p* = .007, *t*_*2*_ (27) = -6.647, *p* < .001]. The subordinate meaning visual targets, on the other hand, were facilitated over the dominant meaning ones in subordinate bias contexts [*t*_*2*_ (27) = 2.051, *p* = .050] but also in dominant bias contexts [*t*_*1*_ (19) = 5.113, *p* < .001, *t*_*2*_ (27) = 10.354, *p* < .001]. Finally, the biasing contexts were not found to facilitate the relevant meaning of the ambiguous word. The unrelated context facilitated the recognition of the dominant meaning as compared to both the dominant bias context [*t*_*2*_ (27) = -6.899, *p* < .001] and subordinate bias context [*t*_*1*_ (19) = -3.320, *p* = .004, *t*_*2*_ (27) = -8.995, *p* < .001], while the dominant bias context facilitated the recognition of the subordinate meaning visual targets as compared to the unrelated context condition [*t*_*1*_ (19) = 4.644, *p* < .001, *t*_*2*_ (27) = 15.532, *p* < .001].

### Interim Summary of LDT data

The across groups analysis revealed ambiguity and context main effects with increased processing time for homonyms and faster response times for the unrelated sentential context followed by the dominant context bias and lastly by the subordinate context bias. A closer look at the adult dataset showed that their performance while unaffected by ambiguity, was driven by frequency and context. The child dataset, on the other hand, exhibited a significant ambiguity effect, since homonymy added processing cost across conditions; frequency appeared to facilitate processing, while no context effects were attested in their performance.

### Accuracy

We turn to the accuracy scores on the recognition of the visual targets per participant group (Table 3).

**Table 3:** Accuracy Scores [%] in Visual Word Recognition (SDs in parentheses)

Sentential Context	Visual Target	Audio Prime	Children	Adults
unrelated context	dominant meaning	Homonym	90 (18)	99.6 (1)
		Control	94.7 (5)	98.9 (2)
	subordinate meaning	Homonym	89.2 (15)	98.8 (1)
		Control	93.5 (10)	99 (1)
dominant meaning context bias	dominant meaning	Homonym	96.6 (4)	99.8 (0.8)
		Control	94.6 (9)	98.6 (1)
	subordinate meaning	Homonym	93.6 (6)	98.4 (2)
		Control	81.2 (33)	99.2 (1)
subordinate meaning context bias	dominant meaning	Homonym	82.4 (29)	99.6 (1)
		Control	96.5 (6)	99 (1)
	subordinate meaning	Homonym	95.1 (4)	99.6 (1)
		Control	94 (9)	98.7 (2)

The analysis showed a main effect of Age [*F*_*1*(1, 38)_ = 7.830, *p* = .008, *η*^2^_*p*_ = 0.171; *F*_*2*(1, 54)_ = 36.416, *p* < .001, *η*^2^_*p*_ = 0.403] with adults outperforming children (adults: 99.1% > children: 91.8%), a main effect of Frequency [*F*_*1*(1, 38)_ = 7.868, *p* = .008, *η*^2^_*p*_ = 0.172] with higher accuracy for dominant visual targets (dominant meaning visual target: 95.9% > subordinate meaning visual target: 95.1%), no main effects of Ambiguity or Context and an interaction among all factors [*F*_*1*(2, 76)_ = 3.500, *p* = .035, *η*^2^_*p*_ = 0.084; *F*_*2*(2, 108)_ = 9.580, *p* < .001, *η*^2^_*p*_ = 0.151].

Additional analysis of the adult data showed a positive priming effect of ambiguity only for dominant meaning visual targets following a sentential context biasing towards the dominant meaning [*t*_*1*_ (19) = 2.854, *p* = .010]. As far as frequency is concerned, accuracy on the dominant meaning visual targets was enhanced over the subordinate meaning visual targets only in dominant bias contexts [*t*_*1*_ (19) = 3.297, *p* = .004, *t*_*2*_ (27) = 2.287, *p* = .030]. No context effects were observed in the adult data.

Further analysis of the child dataset revealed that the ambiguity negatively affected children’s performance since higher accuracy scores were found in control conditions compared to homonym ones [dominant meaning visual target – subordinate context bias: *t*_*1*_ (19) = -2.370, *p* = .029, *t*_*2*_ (27) = -8.360, *p* < .001; dominant meaning visual target – unrelated context: *t*_*2*_ (27) = -2.581, *p* = .016; subordinate meaning visual target – unrelated context: *t*_*1*_ (19) = -2.645, *p* = .016, *t*_*2*_ (27) = -3.224, *p* = .003]. Turning to frequency effects, the subordinate meaning visual targets were facilitated compared to the dominant meaning ones in subordinate [*t*_*1*_ (19) = -2.057, *p* = .054, *t*_*2*_ (27) = -6.793, *p* < .001] but also in dominant biasing contexts [*t*_*2*_ (27) = -6.084, *p* < .001]. On the other hand, context facilitated child visual word recognition. We found that appropriately biasing contexts facilitated accuracy on the favored meaning. More specifically, dominant bias contexts facilitated accuracy on dominant meaning visual targets compared to the unrelated contexts [*t*_*2*_ (27) = -2.738, *p* = .011] and also subordinate bias contexts enhanced accuracy on the subordinate meaning visual targets compared to unrelated contexts [*t*_*2*_ (27) = -2.761, *p* = .010]. Additionally, the subordinate bias contexts negatively affected accuracy on the dominant meaning visual targets compared to the unrelated contexts [*t*_*1*_ (19) = 2.209, *p* = .040; *t*_*2*_ (27) = 3.618, *p* = .001; *t*_*2*_ (27) = -3.261, *p* = .003]. Finally, the dominant bias contexts also facilitated accuracy on the subordinate meaning visual targets as compared to unrelated contexts [*t*_*1*_ (19) = 2.121, *p* = .047, *t*_*2*_ (27) = -9.929, *p* < .001].

### Interim Summary of Accuracy data

The across groups analysis revealed that a main effect of frequency with higher accuracy for dominant meaning visual targets. The adult dataset analysis showed a positive effect of ambiguity and some facilitation of frequency. Meanwhile, the child dataset analysis showed the opposite pattern with regard to ambiguity, since they performed more accurately in control items as opposed to homonyms. Lastly, there was evidence that biasing contexts increased accuracy on the favored meaning.

### Correlation Analyses

We performed correlation analyses in order to explore whether participants’ LDTs and accuracy associate with cognitive screening scores, namely working memory and inhibition. Firstly, we present the outcome of the correlation analyses regarding LDTs. The adult dataset showed no significant correlations, suggesting that adult lexical processing is unaffected by their cognitive resources; note, however, that the adult participants were all highly educated and there was no significant variance among them with regard to their cognitive screening.

The correlation analysis of the LDT child data, nevertheless, showed that their verbal working memory scores related to their processing performance for ambiguity resolution with longer LDTs correlating with lower Digit Backwards Recall scores but only when the primes included homonyms [dominant meaning visual target – dominant context bias: *r* (20) = − 0.624, *p* = .003; dominant meaning visual target – subordinate context bias: *r* (20) = − 0.484, *p* = .031; dominant meaning visual target – unrelated context: *r* (20) = − 0.480, *p* = .032; subordinate meaning visual target – subordinate context bias: *r* (20) = − 0.633, *p* = .003; subordinate meaning visual target – unrelated context: *r* (20) = − 0.512, *p* = .021]. The correlation analysis of inhibitory control and processing lexical ambiguity did not show any significant association between the two but significant correlations were identified for the processing of control items with shorter processing times associated to better inhibitory control performance in some conditions [dominant meaning visual target – dominant context bias: *r* (20) = 0.433, *p* = .056; dominant meaning visual target – unrelated context: *r* (20) = 0.512, *p* = .020; subordinate meaning visual target – subordinate context bias: *r* (20) = 0.473, *p* = .035].

The correlation analysis was followed up by a regression analysis so as to explore the predictive power of the cognitive measures in lexical processing. Verbal working memory scores significantly predicted LDTs for dominant meaning visual targets in the homonym condition (dominant context bias: *ß* = -30.503, *t*(19) = -3.392, *p* = .003, Tolerance = 1.000, VIF = 1.000, *sr* = − 0.624; subordinate context bias: *ß* = -37.773, *t*(19) = -2.347, *p* = .031, Tolerance = 1.000, VIF = 1.000, *sr* = − 0.484; unrelated context: *ß* = -34.963, *t*(19) = -2.324, *p* = .032, Tolerance = 1.000, VIF = 1.000, *sr* = − 0.480) and explained a significant proportion of variance in processing times (dominant context bias: *R*^2^ = 0.390, *F*(1, 18) = 11.507, *p* = .003; subordinate context bias: *R*^2^ = 0.234, *F*(1, 18) = 5.509, *p* = .031; unrelated context: *R*^2^ = 0.231, *F*(1, 18) = 5.402, *p* = .032). Inhibitory control scores significantly predicted LDTs in the control condition (dominant meaning visual target – dominant context bias: *ß* = 66.456, *t*(19) = 2.039, *p* = .056, Tolerance = 1.000, VIF = 1.000, *sr* = 0.433; dominant meaning visual target – unrelated context: *ß* = 65.853, *t*(19) = 2.549, *p* = .020, Tolerance = 1.000, VIF = 1.000, *sr* = 0.515; subordinate meaning visual target – subordinate context bias: *ß* = 69.444, *t*(19) = 2.278, *p* = .035, Tolerance = 1.000, VIF = 1.000, *sr* = 0.473) and explained a significant proportion of variance in processing times (dominant meaning visual target – dominant context bias: *R*^2^ = 0.188, *F*(1, 18) = 4.156, *p* = .056; dominant meaning visual target – unrelated context: *R*^2^ = 0.265, *F*(1, 18) = 6.499, *p* = .020; subordinate meaning visual target – subordinate context bias: *R*^2^ = 0.224, *F*(1, 18) = 5.188, *p* = .035).

We performed a correlation analysis of participants’ accuracy scores across conditions to the cognitive screening scores of working memory and inhibition. The analysis showed no significant correlations suggesting that lexical access itself is not affected by cognitive skills.

### Interim Summary of Correlation Analyses

The correlation and regression analyses showed that adults’ lexical processing and accuracy was unaffected by their cognitive skills. Note however, they all had very similar cognitive skills. Similarly, the child correlation of accuracy scores did not reveal any relationship to cognitive capacity but the correlation analyses of their processing times did show a close relationship between (a) verbal working memory skills and the processing of homonyms and (b) inhibition and the processing of control items. These findings were also verified by the respective regression models. Overall, cognitive capacity resources appear to contribute to lexical ambiguity resolution when looking at developing language data but not when examining a stable language system in relation with cognitive skills verifying earlier research.

### Visual Non-Word Recognition & Processing

With regard to the non-word items of the cross-modal priming task that support the lexical decision component of the task, we analyze the performance of participants in relation to the status of non-words as illegal or pseudo-words due to the fact that it informs us on how the mental lexicon functions providing evidence on the lexical identification skills of the participants and the lexical access process when no lexical entry is available. Table 4 shows the accuracy and the LDTs in the visual non-word recognition per condition for both groups.

**Table 4:** Visual Non-Word Recognition & Processing (SDs in parentheses)

Measurement	Non-words	Children	Adults
Accuracy (%)	Illegal	97.6 (4)	99.7 (0.4)
	Pseudo	89.8 (12)	96.7 (1)
Processing (LDTs in msec)	Illegal	1186 (267)	599 (98)
	Pseudo	1478 (270)	704 (150)

The data analysis suggests that both children and adults are better and faster at identifying illegal items as non-words in Greek [Accuracy – children: *t*_*1*_ (19) = 4.197, *p* < .001, *t*_*2*_ (59) = 7.001, *p* < .001; Accuracy – adults: *t*_*1*_ (19) = 10.018, *p* < .001, *t*_*2*_ (59) = 3.082, *p* = .003; LDTs – children: *t*_*1*_ (19) = -9.936, *p* < .001, *t*_*2*_ (59) = -14.677, *p* < .001; LDTs – adults: *t*_*1*_ (19) = -7.626, *p* < .001, *t*_*2*_ (59) = -17.936, *p* < .001].

Next, the correlation analysis of visual non-word recognition and the cognitive screening measures showed there is a correlation only of processing time to verbal working memory scores in the child dataset with shorter LDTs relating to higher scores in the Digit backwards task [illegal: *r* (20) = − 0.550, *p* = .012; pseudo: *r* (20) = − 0.531, *p* = .016]. The follow-up regression analysis showed that verbal working memory scores significantly predicted LDTs for both non-word types (illegal: *ß* = -31.935, *t*(19) = -2.797, *p* = .012, Tolerance = 1.000, VIF = 1.000, *sr* = − 0.550; pseudo: *ß* = -31.088, *t*(19) = -2.656, *p* = .016, Tolerance = 1.000, VIF = 1.000, *sr* = − 0.531) and explained a significant proportion of variance in LDTs (illegal: *R*^2^ = 0.303, *F*(1, 18) = 7.821, *p* = .012; pseudo: *R*^2^ = 0.281, *F*(1, 18) = 7.052, *p* = .016). Similarly to the lexical ambiguity resolution data, cognition appears to contribute to non-word recognition when examining developmental data.

### Interim Summary of non-word data

The non-word data analysis showed that illegal non-words are processed faster and more accurately compared to pseudo-words both by adult speakers and children. The correlation analysis of the non-word data and cognitive skills revealed that in the case of child data alone processing time of non-words and verbal working memory skills were closely related with better cognition skills boosting the performance of children.

## Discussion

The study set out to examine lexical access in Greek ambiguous words so as to identify the contribution of frequency and context in ambiguity resolution (Research Question (a) for developing and stable grammars (Research Question (b). To follow the course of meaning activation and selection, we opted for homonyms which due to their constant phonological and orthographic cues can help us disentangle the role of meaning frequency and context in ambiguity resolution. Earlier studies on lexical ambiguity resolution have identified sentential context and word frequency as key features that can affect lexical processing (Chen & Boland, 2008; Vu et al., 2000). Studies focusing on the performance of adults emphasize the frequency of alternative meanings as well as the interaction between contextual bias and frequency (Binder & Rayner, 1998; Chen & Boland, 2008; Vu et al., 1998); in particular, while processing unbiasing sentential contexts the most frequent, dominant meaning of an ambiguous word was found to be activated more quickly than less frequent, subordinate meanings (Dopkins et al., 1992; Lucas, 1999; Sereno, Brewer, & O’Donnell 2003; Simpson & Krueger, 1991). For younger speakers, on the other hand, sentential context appears to have an incremental effect in performance in late childhood while lexical-level effects such as frequency seem to decrease with age (Booth et al., 2006; Gernsbacher et al., 1990). Overall developmental data suggest that there are difficulties with regard to top-down processing with the difficulty to integrate linguistic context still being evident at the age of 12.

To accommodate this evidence, the current study tested both children and adults so as to provide a clearer outline of the monolingual mental lexicon. In addition, word–meaning association strengths in ambiguous words may vary significantly, and consequently processing can be costlier for linguistic context integration tasks often reported as a strong subordinate bias effect (Balota et al., 2001; Binder, 2003; Novick et al., 2005; Rayner et al., 2006; Sereno, O’Donnell, & Rayner 2006). In order to avoid this type of effect, the methodological design cross-checked the frequency of all relevant meanings of the homonyms tested and selected relatively equibiased items with a high frequency dominant meaning (74.8%) and a low frequency subordinate meaning (38.4%) excluding polarized items and ambiguous items with the same distribution of both meanings.

The data analysis of accuracy scores in visual word recognition showed a main effect of frequency in the dataset with dominant meaning visual targets being more accurately identified than subordinate meaning visual targets which is in line with earlier literature on lexical ambiguity resolution (Chen & Boland, 2008; Vu et al., 2000). Adults’ performance did not appear to be much regulated by ambiguity or context since their visual word recognition was rather intact with accuracy scores higher than 98% across experimental conditions. Further exploration of the child dataset showed that context interplayed with frequency during visual word recognition. Firstly, the accuracy on visual targets whose meaning was favored in the preceding context was facilitated compared to the unrelated context. Secondly, we found evidence that the context inhibited the non-favored meaning, as the subordinate bias context negatively affected accuracy of dominant meaning visual targets compared to the unrelated context. Notice, however, that performance on subordinate meaning visual targets was enhanced by both dominant and subordinate meaning biasing contexts as compared to unrelated contexts. This finding indicates that the children encounter some difficulties in effectively incorporating the sentential context cues during lexical ambiguity resolution, as reported in previous studies (Booth et al., 2006), particularly when word recognition involves the less frequent meaning of the homonym. The evidence with regard to accuracy of lexical access appears to verify our research hypothesis with regard to the child data and only partly with regard to the adult dataset who we anticipated would be more sensitive to frequency and context variables.

Turning to the adult processing data, the analysis did not show strong lexical priming effects. The adults’ performance on visual targets appears to be highly controlled and overall unaffected from ambiguity, with priming emerging only in two conditions; a facilitation effect for dominant meaning visual targets in unrelated contexts and an inhibition effect for dominant meaning visual targets in the dominant meaning bias contexts and for subordinate meaning visual targets in subordinate contexts. The former result suggests that when sentential integration is not demanding, as in unrelated contexts, homonyms can facilitate high frequency meaning targets, whereas when sentential context and targets match both meanings of homonyms remain active and have an inhibition effect. This evidence provides support to our hypothesis that the interaction of frequency and contextual bias can be critical in the processing of ambiguity by adult native speakers and not the independent contribution of context per se. Andreou et al. (2009), who tested adult native speakers of Greek, also reported limited priming effects with regard to context. In the case of our dataset, the highly automated processing of our adult participants can be attributed to the fact that they were highly qualified speakers with an average of 19.1 years of education; their skilled reading could cover the processing effects attested in earlier studies (see Duffy et al., 2001; Kellas et al., 1995 among others). However, considering the role of literacy skills in processing it is not surprising that the adult participants could outweigh linguistic information integration effects (see Gernsbacher & St. John, 2001; Rabagliati et al., 2013; Snedeker, 2013; Snedeker & Yuan, 2008; Trueswell & Gleitman, 2007). Nevertheless, this assumption requires confirmation with further lexical processing data to be obtained from speakers of different literacy profiles so as to be able to explain fully the impact of language external factors.

The analysis of the adult processing times showed an advantage for the dominant meaning in unrelated contexts, which is in line with previous work (Andreou et al., 2009). However, we found advantages for the dominant and the subordinate meaning visual targets in the contexts that are not biased towards them; the dominant is favored over the subordinate one in dominant bias contexts and the subordinate meaning in dominant bias contexts. It seems that the biasing contexts enhanced facilitation of the non-favored meaning. Notice, also, that the adult LDT data do not show evidence for context effects, in that dominant and subordinate meaning visual targets are facilitated in unrelated rather than in biasing contexts. These findings are difficult to accommodate within current lexical ambiguity resolution literature. One possible explanation that we would like to put forward relates to the high cognitive capacities and reading skills of our adult participants, which may have contributed to the suppression of the contextually favored meaning. A second explanation is a methodological one. More specifically, the ISI in this study was set at 0ms, due to previous findings in Greek (see Andreou et al., 2009). This short ISI interval, may, however, have impeded the emergence of contextual effects. We leave this issue open for future investigation.

The children’s processing times for ambiguity resolution showed a strong ambiguity effect with homonyms processing being highly costly along with a main effect of context with faster LDTs for unrelated context and slower LDTs for subordinate context bias (unrelated context < dominant context bias < subordinate context bias). Frequency was also a significant factor in ambiguity resolution; specifically, for unrelated contextual primes faster response times were found for dominant meaning visual targets as opposed to subordinate meaning visual targets. Moreover, as attested in the adult data, the biasing contexts did not facilitate the processing of the ambiguous words, since on one hand the unrelated context primed the dominant meaning more effectively than the dominant and the subordinate bias contexts and on the other hand the dominant bias context enhanced the subordinate meaning compared to the unrelated context. These two findings corroborate the child accuracy data of our study and provide further support for the integration difficulties that children even in late childhood are still facing verifying our research hypothesis (Booth et al., 2006; Gernsbacher et al., 1990).

Lastly, both for children and adults’ cognitive resources are critical in lexical processing. Prior research has shown that the children’s difficulties to engage in top-down processes is related to the availability of cognitive resources required to process language (Coch & Holcomb, 2003; Khanna & Boland, 2010). In view of the working memory requirements for lexical access, we included screening of working memory and inhibition which have been identified as critical for top-down processing (for detailed discussion see Bunge et al., 2002; Casey et al., 2005; Protopapas et al., 2007). The analysis revealed that decision making in lexical access is not related to the cognitive resources available either in the child or adult dataset. There were though significant correlations when we examined the LDTs of children. In the case of homonyms and non-words better verbal working memory skills were correlated to faster processing and in the case of control unambiguous items better inhibition skills facilitated language processing. An intriguing finding is that working memory and inhibitory control differentially affect lexical processing. Working memory is involved in the recognition of visual targets whose meaning has been primed by an ambiguous word but also by preceding contextual information. It seems, thus, that the capacity of working memory has a significant impact on word recognition when the retainment of various information sources, such as contextual meaning, contributes to faster lexical processing. Τhe processing of control words, by contrast, demands inhibitory control skills, since preceding lexical and contextual cues need to be suppressed for the recognition of the control words to be fast and efficient. Overall, the correlations attested in our child data between lexical processing and cognitive skills suggest that for developing grammars of 10;4 to 11;3 yrs old children the automatic spreading of activation within the semantic network is supported by cognitive skills such as working memory thus verifying our research hypothesis. The adult data, though, did not exemplify this effect due to the high educational background of the participants and the homogeneity in performance in their cognitive screening.

To conclude, with regard to Research Question (a) our adult data indicate that meaning frequency has a significant impact on lexical ambiguity resolution, while the context effects were limited, and, thus argue in favor of accounts in lexical processing which prioritize frequency over contextual cues (Kellas et al., 1995; Vu et al., 2000, 2003). The child data, on the hand, display a somewhat different picture, since contextual information does not facilitate word recognition. The child lexical processing system appears to be more affected by frequency than by sentential context and, thus, displays a bottom-up pattern. Finally, in relation to Research Question (b), the processing of ambiguous words is costly for children and affected by working memory and inhibitory skills.

## Footnotes

1 With regard to the modality of the tasks, most studies present the ambiguous word in written form apart from a few studies (e.g. Chen & Bolland, 2008; Friedrich & Friederici, 2005; Henderson, Clarke & Snowling, 2011).

2 Note that the primary focus of the Andreou et al. (2009) study is to examine the effects of sentential context on lexical ambiguity resolution in patients with schizophrenia; hence, only the data of the non-clinical population is referred to with this section.

3 ISI in experimental design refers to the temporal interval between the offset of one stimulus to the onset of another.

4 In a congruent relationship both the context and the target related to the same meaning, while in an incongruent relationship context and target related to different meanings.

5 Top-down parsing models prioritize context and background knowledge in explaining language processing, while bottom-up parsing models rely on semantic, syntactical and phonological properties to describe the process (see detailed discussion in Rabagliati et al., 2013).

6 Note that the sample size in this pre-task was increased to ensure that we have identified the appropriate target items with word length and frequency conditions also satisfied.

7 Ethical consideration was given to all aspects of experimental study and written informed consent from legal guardians of minors was obtained. Ethics approval was granted by the AUTH Ethics Committee No. 212201/2021.

8 Out of the 30 homonyms, 2 words were excluded from the analysis due to an audio tech issue.
